# Quantitative proteomic analysis of bile in extrahepatic cholangiocarcinoma patients

**DOI:** 10.7150/jca.40964

**Published:** 2020-04-07

**Authors:** Kuk Hui Son, Chi Bum Ahn, Hyo Jung Kim, Jae Seon Kim

**Affiliations:** 1 Department of Thoracic and Cardiovascular Surgery, Gachon University Gil Medical Center, Gachon University, Incheon, 21565, Republic of Korea; 2 Center for information security technologies, Korea University; 3 Department of Internal Medicine, Korea University Guro Hospital

**Keywords:** Cholangiocarcinoma, Bile, Proteins

## Abstract

**Background and Aims**: Extrahepatic cholangiocarcinoma (CCA) without liver-fluke is increasing. Multifactorial carcinogenesis makes it hard to find biomarkers related to CCA. Although there are a few studies of bile proteomics, these showed different protein profiles because of having heterogeneous groups of patients and different sampling methods. Our aim was to identify the specific bile proteins of extrahepatic CCA patients.

**Methods**: We collected bile from 23 patients undergoing endoscopic nasobiliary drainage in Korea University Guro Hospital from May 2018 to January 2019. The CCA group included 18 patients diagnosed with extrahepatic CCA, and the control group included 5 patients with benign biliary conditions. We analyzed bile proteome using liquid chromatography mass spectrometry. We compared the relative abundance of various proteins in the CCA and control groups.

**Results**: In all, we identified a total of 245 proteins in the bile of CCA and control patients. Increased top 14 proteins in CCA patients were immunoglobulin kappa light chain, apolipoprotein B, inter-alpha-trypsin inhibitor heavy chain H4, apolipoprotein E, Mucin 5B, inter-alpha-trypsin inhibitor heavy chain H1, apolipoprotein A-IV, intercellular adhesion molecule 1, complement C7, complement C5, apolipoprotein C-III, albumin, antithrombin-III, and apolipoprotein A-II. However, the significantly increased proteins in bile of CCA patients comparing with control patients were immunoglobulin kappa light chain, apolipoprotein E, albumin, apolipoprotein A-I, antithrombin-III, α1-antitrypsin, serotransferrin, immunoglobulin heavy constant mu, immunoglobulin J chain, complement C4-A, and complement C3 (p<0.05).

**Conclusions**: In this study, we identified several proteins that were significantly increased in the bile of extrahepatic CCA. Further study is needed to validate them as potential tumor-associated proteins that may be potential biomarkers for CCA.

## Introduction

Cholangiocarcinoma (CCA) is a lethal malignant tumor with dismal outcomes; diagnosis in its early stage is very important. The diagnosis of CCA, especially when CCA is accompanied with extrahepatic bile-duct stricture, is very difficult, even using blood tests, imaging studies, and endoscopic methods. Although the direct tissue sampling using endoscopic retrograde cholangiopancreatography (ERCP) is a proper diagnostic tool, there are some limitations for achieving adequate sample 1.

Several studies have investigated the possibilities of liquid biopsy using blood, bile juice, sweat, urine, and feces as a novel and noninvasive diagnostic method 2-5. Cancer-derived protein can be released into bile by necrosis and apoptosis of tumor cells, and bile includes tumor-specific molecules and provides useful information during malignant transformation in real time 5,6. Use of proteomics to detect biomarkers in bile may hold promise in aiding differentiation of malignant from benign biliary strictures. To date, a few studies have investigated the proteins in bile. Despite improvements in bile analysis, a promising diagnostic biomarker remains unknown, because the results were not consistent. Besides the diversity of bile proteins, there are the diversity of bile sampling, enrolled patients, and the anatomic location of CCA.

Thereafter, we explore the comparative bile analysis of patients with extrahepatic CCA and control patients without bile-duct disease.

## Materials and Methods

### Patients

Patients who had undergone ERCP with endoscopic nasobiliary drainage (ENBD) were enrolled in Korea University Guro Hospital from May 2018 to December 2019. The inclusion criterion was age > 18 years.

The patient group was 18 patients who had extrahepatic CCA based on histologic diagnosis. Patients with gallbladder or ampullary caner were excluded.

The control group was 5 patients who had undergone ENBD without biliary disease. They needed bile drainage for bile-duct injury after a hepatobiliary operation (2 patients) or benign ampullary stenosis (3 patients).

In both groups, we excluded patients with symptoms of cholangitis or turbid bile on visual exam and patients who could not have a normal diet. All recruited patients had at least six months follow-up to exclude the possibility of misdiagnosis as malignancy of a benign condition in three patients who had benign ampullary stenosis.

Informed consent was obtained from all patients. This study was approved by the Ethics Committee at Korea University Guro Hospital (approval number 2018GR0133).

### Bile sampling

We obtained approximately 5 mL of fresh bile through the ENBD tube after drainage of stagnant old bile, about two or three days after the ERCP procedure. On same day, we obtained blood tests and clinical information. The collected bile samples were directly frozen at -80°C until analysis.

## Experimental procedures

### (1) Sample preparation

A bile sample of 1 mL was centrifuged at 10,000 x g and 4°C for 15 minutes. We mixed the supernatant collected after centrifugation with 9 mL of ice-cold methanol. The mixture was incubated overnight at -20°C. After confirming the precipitate, we centrifuged the sample at 14,000 x g, 4°C for 30 minutes and the supernatant was removed. After we repeated the centrifuging of the sample and removed of the supernatant, the pellet was dried in the air, then dissolved in lysis buffer (20 mM Tris-Cl (*p*H 8.0), 150 mM NaCl, 2 mM EDTA, 10 % Glycerol, 1 % NP-40). We then measured its protein concentration with the Bradford Assay.

An aliquot of 100 ul (100 μg of lysate resuspended with 50 mM ammonium bicarbonate solution) was reduced with 10 mM DL-Dithiothreitol Solution by shaking for 1 hour at 37°C and was alkylated with 50 mM Iodoacetamide at room temperature and was shaken for 1 hour in the dark. To reduce the Dithiothreitol concentration to a final 1 mM with 1.000 μl volume, we added the 50 mM ammonium bicarbonate. The sample was then digested with trypsin at a protein-to-enzyme ratio of 40:1 at 37°C overnight.

To quench the digestion reaction, we added 1% trifluoroacetic acid (TFA). We used Waters Oasis MCX (Waters, Irand, Waters cat No. 186000252) cartridge to clean up the sample (MCX column work). The sorbent was equilibrated by adding 1 mL of methanol, and then washed with 1 mL of 0.1 % TFA. After adding 500 to 1,000 μl of acidified sample (< *p*H 2) to load, we washed it with 1 mL of 0.1 % TFA and 1 mL of methanol. The sample was eluted with 500 to 1,000 μl of elution buffer (50 % acetonitrile, 5 % Ammonium hydroxide). The eluate was dried to completion in a Speedvac (Thermo Fischer Scientific, San Jose, CA, USA), and then dissolved in 0.1 % formic acid for LC injection or stored at -20°C before the analysis.

### (2) Liquid chromatography-mass spectrometry (LC-MS) configuration

We did LC analysis using a Thermo Scientific Eazynano LC II autosampler with a reversed-phase peptide trap EASY-Column (100 μm inner diameter, 2 cm length) and a reversed-phase analytical EASY-Column (75 μm inner diameter, 10 cm length, 3 μm particle size). The extract was injected in 1/10 volumes, and we did LC using 0.1% formic acid in water as buffer (A) and 0.1% formic acid in acetonitrile as buffer (B), at a flow rate of 300 nL/min.

Collision-induced dissociation (CID)-type fragmentations were activated by Automatic data-dependent LTQ-Orbitrap (20 MS2 (LTQ) + 1 Full MS (Orbitrap)) switching modes. The MS source conditions included the capillary voltage optimized to 1.9 kV, at 275°C, range 300 - 2,000 m/z. Resolution was 100,000. The dynamic exclusion with a repeat count of 1, exclusion duration of 180 seconds, list size of 300 activated.

### (3) Mass spectrometry data analysis system

Each processed databit was subsequently transformed to the Sorcerer 2 and Scaffold 4 program. The search parameters used were as follows: 25 ppm tolerance for precursor ion mass and 1.0 Da for fragment ion mass. Finally, protein and peptide were displayed with threshold 80 %, minimum peptides 2, and quantified by Top 3 Total Ion Chromatogram (minimum value =0.01, Normalization) method. We also did protein classification based on functional annotations using Gene Ontology for molecular function, biological processes, and cellular component categories.

## Results

We analyzed a total of 23 bile samples, from the 18 patients with extrahepatic cholangiocarcinoma and the 5 patients with benign biliary conditions in the control group. The baseline characteristics of the enrolled patients are summarized in Table 1.

### Malignant vs. benign condition

Each of the 23 bile samples was processed by LC-MS analysis for protein identification and quantification.

**Figure 1:**
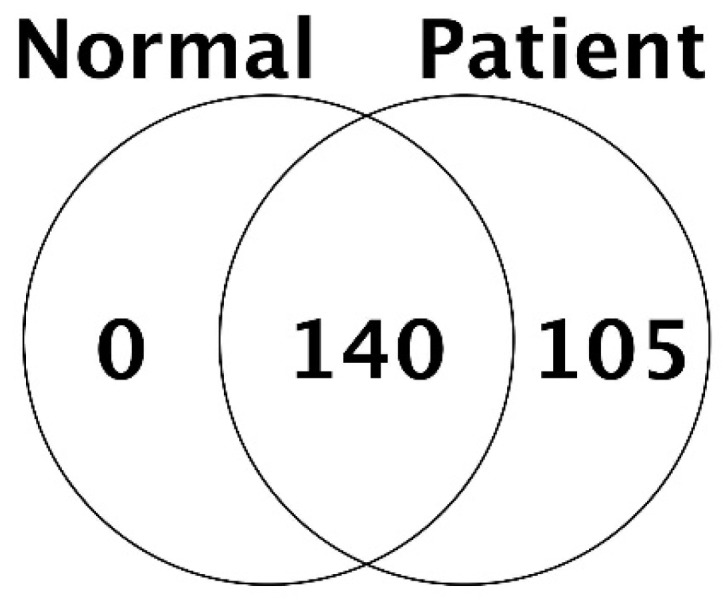
Number of proteins identified in control (normal) and cancer patients

In all, we identified a total of 245 proteins in the bile of CCA patients and control patients. The number of common protein between control and CCA patient was 140, and 105 of protein was confirmed only in bile of CCA patients (Fig. 1).

The increased proteins with spectral counts of more than twofold were immunoglobulin kappa light chain, apolipoprotein B, inter-alpha-trypsin inhibitor heavy chain H4 (ITIH4), apolipoprotein E, Mucin 5B, inter-alpha-trypsin inhibitor heavy chain H1 (ITIH1), apolipoprotein A-IV, intercellular adhesion molecule 1, complement C7, complement C5, apolipoprotein C-III, albumin, antithrombin-III, and apolipoprotein A-II (Table 2).

**Figure 2:**
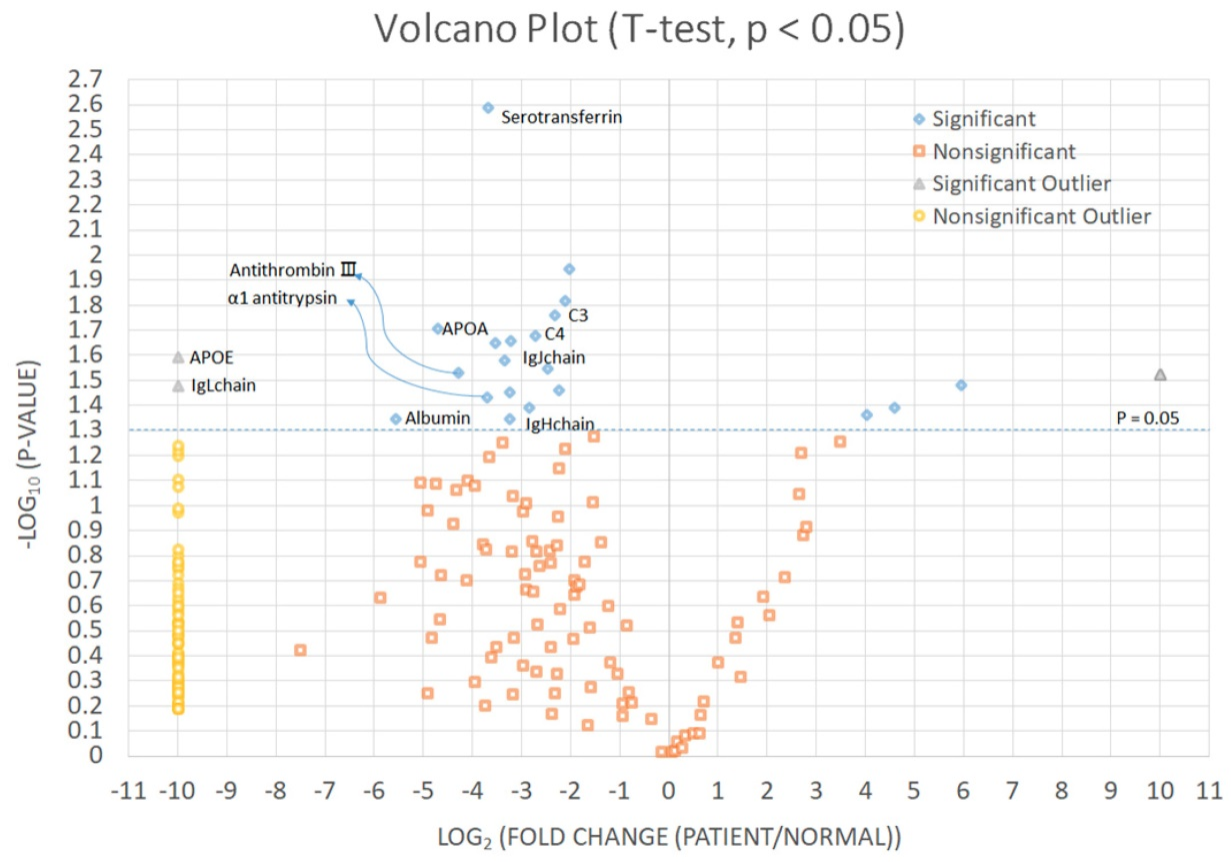
Significantly increased proteins in cholangiocarcinoma patients

We identified significantly increased proteins in CCA patients than in the control group (Fig. 2 and Table 3). Immunoglobulin kappa light chain, apolipoprotein E, albumin, apolipoprotein A-I, antithrombin-III, α1-antitrypsin, serotransferrin, immunoglobulin heavy constant mu, immunoglobulin J chain, complement C4-A, and complement C3 were significantly increased in bile of CCA patients comparing with control patients.

### Bile of patients with CCA

**Figure 3:**
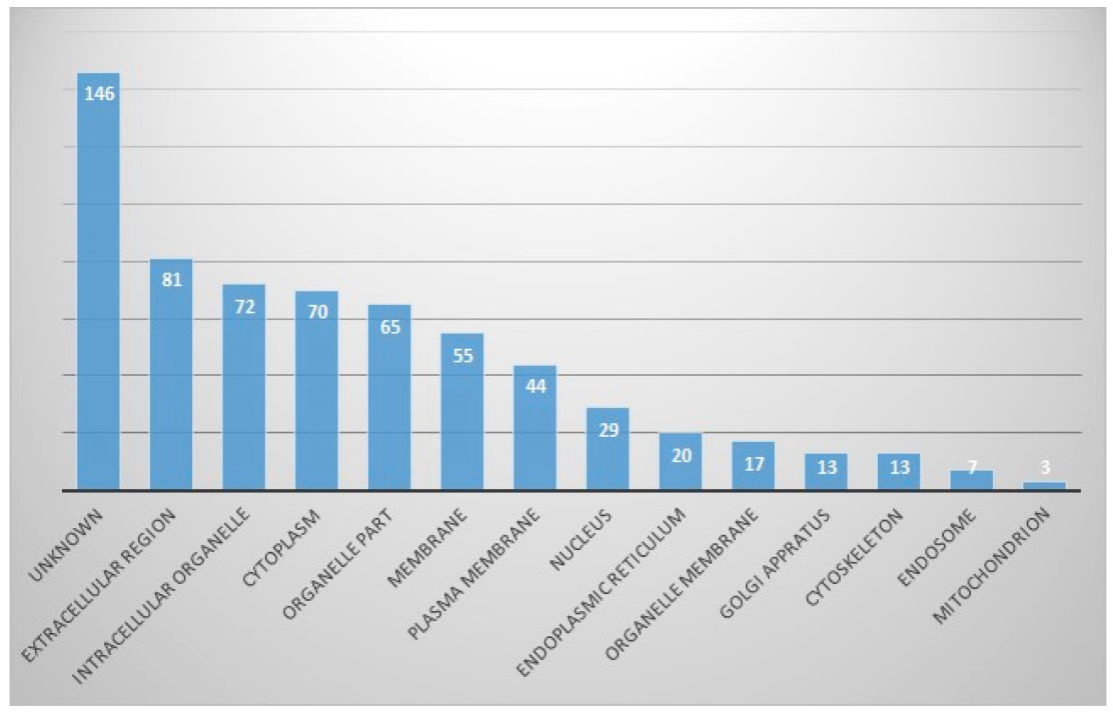
In cellular component part, proportion of identified proteins in bile

In the cellular component part, proteins originating from the extracellular region were the most common (13%), followed by the intracellular organelles (11%) and cytoplasmic part. Other identified proteins were from organelle parts, membranes, plasma membranes, nuclei, endoplasmic reticula, and so on. However, proteins of unknown origin were considerably high, at 14.2% (Fig. 3).

**Figure 4:**
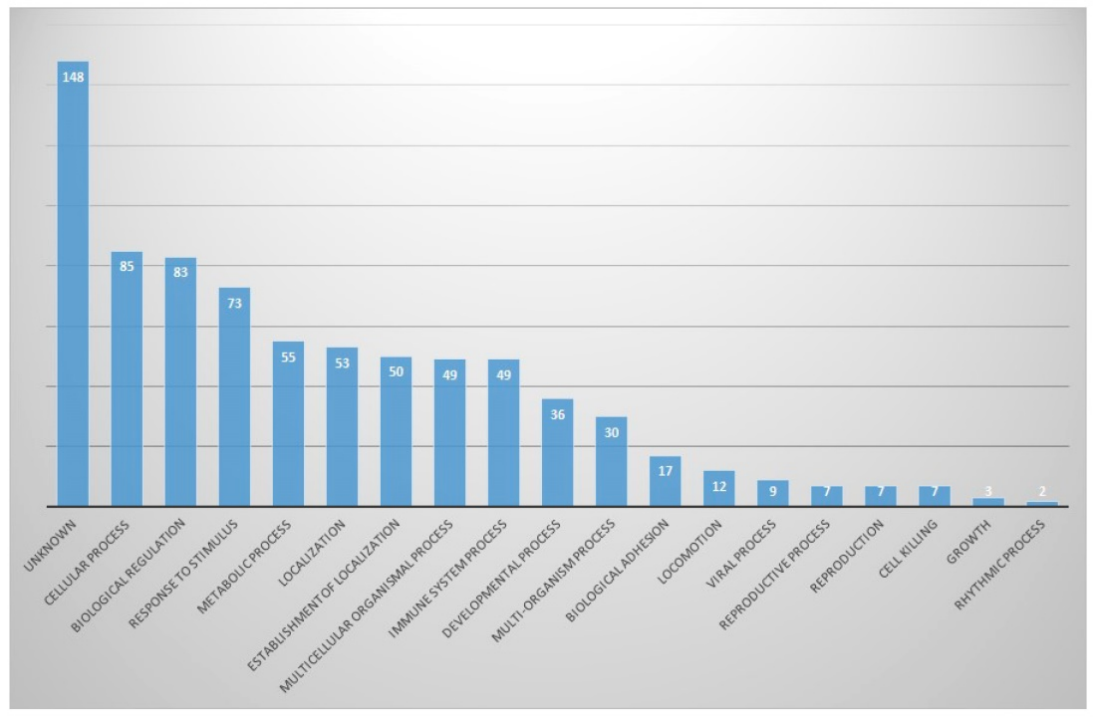
In biological process part, proportion of identified proteins in bile

In biological aspects, proteins involved in cellular processes (11%) and biological regulation (10.7%) were the most abundant, and proteins involved in response to stimuli, metabolic process and localization were next. There were also many unknown proteins (19.0%) (Fig. 4).

**Figure 5:**
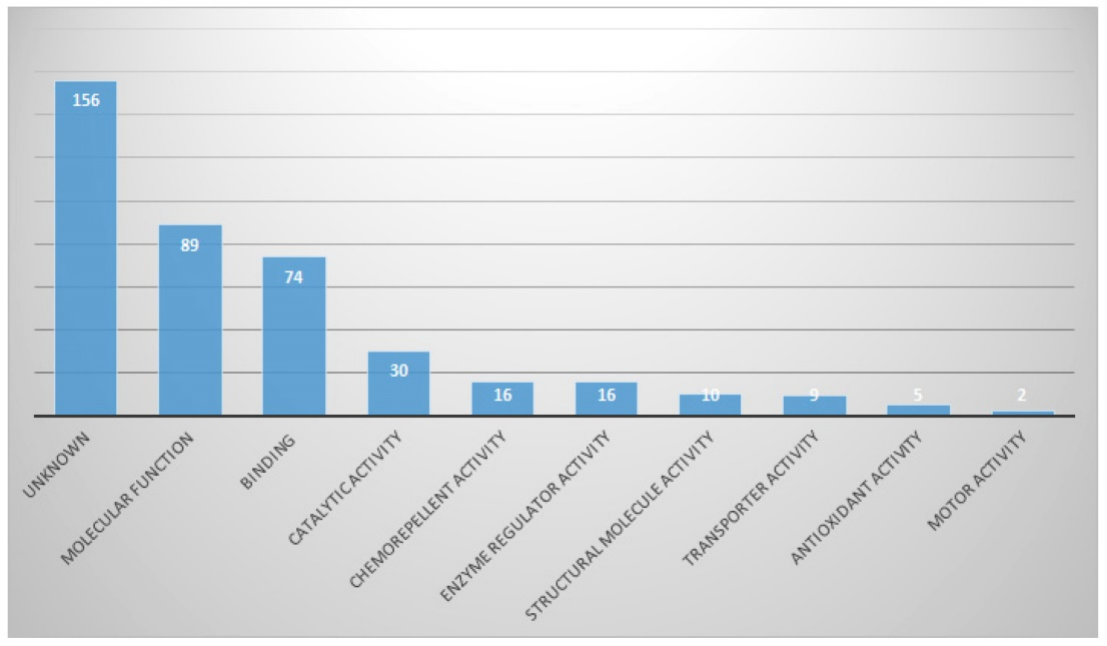
In Molecular function part, proportion of identified proteins in bile

Proteins with molecular functions were the most abundant (21.9%), and then proteins in categories of binding and catalytic activities (Fig. 5).

## Discussion

Tissue sampling such as cytology or biopsy through ERCP is not enough. The sensitivity for malignancy diagnosis by ERCP with radiologic evaluation and biopsy is only 9-57% 7,8.

Bile analysis for diagnosis of CCA is a promising method, especially in differentiation of biliary stricture, because CCA which originates from the biliary epithelium might release proteins from cancer cells into bile 9. Thus, proteomic analyses of bile for discovering CCA biomarkers have been done in many studies 9-15. However, the bile-protein profiles of CCA were different from the studies. One reason for those differences is the diversity of CCA types enrolled in studies. Previous studies enrolled both of intrahepatic and extrahepatic CCA in the same study. CCA is a heterogeneous entity and is defined by anatomic location as intra or extrahepatic CCA 15. For intrahepatic CCA, the different characterization depended on whether the liver is cirrhotic or not 15. Thus, the tumor character differences by anatomical location might affect the bile-protein profile. One study demonstrated that intrahepatic CCA is a heterogeneous group affected by background liver conditions on tumor biology, while extrahepatic CCA is a more homogeneous subgroup 15. For elimination of heterogeneity of cancer tissue, we enrolled only extrahepatic CCA in our study.

Previous studies of bile proteome analysis for CCA biomarkers were usually based on comparison between CCA and control groups. The control group showed diversity in the studies and contained various disease entities, such as benign biliary conditions, choledocholithiasis, and even primary sclerosing cholangitis (PSC) 9-15. It has been known that proteins associated with the Wnt signaling pathway were highly expressed in CCA without PSC 16,17. However, pathogenesis of PSC-associated CCA is more complex, and pro-inflammatory cytokines might have an important role 18. One study of bile proteomic analysis showed that the most elevated pathways in PSC-associated CCA were inflammation-associated cytokine and chemokine pathway; however, the Wnt signaling pathway was mainly elevated in CCA without PSC 14, which suggested that enrolled patient diversity in the control group might affect the diversity of the bile-protein profile in the studies. Thus, we enrolled only benign biliary conditions that needed bile drainage and were not induced by biliary disease. Those patients seemed to be closer to a normal control.

Bile is produced by the hepatocytes and is secreted into bile ducts. It is stored in the gallbladder, which concentrates hepatic bile, hence the bile profile might be different from the collecting site of the biliary tree. Previous studies used concentrated bile from the gallbladder or common bile duct during cholecystectomy or ERCP, however, we used fresh secreted bile instead of concentrated bile in our study in order to avoid the change of bile profile during concentration or staying in the gallbladder.

Apolipoprotein E is involved in DNA synthesis, cell proliferation, angiogenesis, and metastasis, and changes of these functions might induce tumorigenesis or progression 19.

Apolipoprotein E overexpression has been reported in various cancers, such as gastric, lung, and prostate 20-22. In our study, apolipoprotein E and apolipoprotein A-I in the bile of CCA were significantly increased. Apolipoprotein A-I is synthesized in the liver and small intestine 23. Several studies have suggested that the serum level of apolipoprotein A-I could be a potential biomarker for CCA 24. Apolipoprotein has a key role in lipoprotein metabolism but it's role in bile is not well known. It is presumed that apolipoproteins transport lipid in bile as in the serum, and that apoproteins are transported in the hepatocytes as vesicles and released into the bile across the canalicular membrane 25. It is suggested that release of apolipoprotein into bile could be increased according to the change of bile lipid in cholangiocrcinoma.

Antithrombin is known as a protein related with hemostasis, but it has been studied as an anti-tumor effect through inhibiting a protease involved in metastasis and generating an anti-angiogenic molecule 26. It was reported as a modulator of tumor cell migration and invasion in gastric cancer 27. And the novel function as a diagnostic biomarker was studied in hepato-pancreato-biliary malignancy 28, 29. In our result, antithrombin-III was significantly increased in the bile of CCA.

α1-antitrypsin is a glycoprotein produced by hepatocytes and mononuclear phagocytes, and is the principal human inhibitor of neutrophil elastase 30. α1-antitrypsin was expressed in the tumors of CCA patients 31. One study that analyzed the bile of six CCA patients showed α1-antitrypsin was an overexpressed protein and might be a marker for diagnosis of CCA 9. In our study, α1-antitrypsin was elevated more than in the control group.

Serotransferrin is serum transferrin synthesized in the liver. Previous studies found that iron metabolism and iron regulatory proteins were altered in cancer and transferrin receptor-1 was increased in cholangiocarcinoma 32,33. Changes in glycosylation of serotransferrin occur in CCA patients, and these glycoforms could be used as risk biomarkers and prognosis and diagnosis markers of CCA 34. In our study, the level of serotransferrin was increased in CCA.

Liver has Kupffer cells and several immune cells and plays an important role in immune response 35. Immunoglobulin A is known as an abundant protein in normal bile, contributes to immunological surveillance within the biliary system 36. Clinically, increase in serum immunoglobulins are observed in specific hapatobiliary diseases such as autoimmune hepatitis (elevated IgG), primary biliary cirrhosis (elevated IgM) and alcoholic liver disease (elevated IgA), Immunoglobulin G4-related sclerosing cholangitis (elevated IgG) 36. It has not been known whether immunoglobulins are increased in bile of biliary tract cancer. In our study, immunoglobulin kappa light chain showed the greatest change between the CCA and control groups. In addition to Immunoglobulin, complement proteins were reported to participate in local immune response in the biliary tract 35. A recent study of plasma proteins analysis found that complement C3 and apolipoprotein C-III were essential proteins in HCC 37. Our results showed that complement C4 and C3 were significantly elevated in CCA patients. Another study of bile proteomic analysis that distinguished CCA from PCS showed that ITIH4 was elevated in CCA 9, as is similar to our study but it was not significant. ITIH4 is a serum glycoprotein secreted mainly by the liver 38-40, and involved in stabilization of the extracellular matrix 40,41. A previous study showed that ITIH4 plays a significant role in the pathway involved in TGF-β and fibrosis-independent carcinogenesis 38, 42. In a study of pancreatic cancer biomarkers using pancreatic juice samples, potential biomarkers complement C5 and ITIH3 were elevated in cancer, but biliary obstruction had a significant effect on the performance of the markers 42.

Albumin is produced solely by hepatocytes and relatively abundant protein in bile. It was founded that the neonatal Fc Receptor is required for delivery of synthesized albumin to the blood stream and absence of this receptor results in increased albumin levels in the bile 43. Cholangiocyte is differentiated from liver progenitor cells and alterations in bile secretion and abnormal bile composition can result in hepatocellular and/or bile duct injury 44,45.

Cholangiocarcinoma cell arising from cell proliferation after bile duct injury could be a form of undifferenciated cholangiocyte producing albumin. There are several reports on aberrant expression of albumin; it could be used to distinguish bile duct neoplasm from other metastatic adenocarcinoma 46-48.

In conclusion, we identified the bile proteins that were elevated in the extrahepatic CCA more than in the control group, who did not have a biliary disease, and the significantly increased proteins were immunoglobulin kappa light chain, apolipoprotein E, albumin, apolipoprotein A-I, antithrombin-III, α1-antitrypsin, serotransferrin, immunoglobulin heavy constant mu, immunoglobulin J chain, complement C4-A, and complement C3. Even though further study is needed, those proteins in bile have potential as biomarkers of CCA.

## Figures and Tables

**Table 1:** Patient characteristics

Number	Pathology	Age (yrs)	Sex	Albumin (g/dL)	Bilirubin (mg/dL)	AST (IU/L)	ALT (IU/L)	CA 19-9 (U/ml)
1	Control	70	F	3.6	0.32	27	30	
2	Control	33	F	4.5	0.55	64	55	
3	Control	59	F	4.1	0.37	16	10	
4	Control	67	F	3.6	2.04	41	55	
5	Control	60	M	3.6	0.35	22	22	
6	Hilar CC	67	M	3.7	13.6	70	185	320
7	CBD ca	47	F	4.1	1.96	135	170	0.4
8	CBD ca	58	M	3.0	5.24	51	69	255
9	CBD ca	72	M	2.9	12.3	111	112	960
10	CBD ca	77	M	3.3	0.48	36	23	27
11	CBD ca	72	F	3.3	0.85	22	18	42
12	CBD ca	75	M	3.5	1.35	141	127	50.6
13	Hilar CC	56	M	3.9	24.5	32	46	12.8
14	CBD ca	70	F	3.3	7.34	93	88	476
15	CBD ca	75	F	3.3	0.41	26	81	20.5
16	Hilar CC	65	M	4.2	0.89	19	59	36.6
17	Hilar CC	83	F	2.7	24.3	118	75	950
18	CBD ca	79	M	3.9	4.19	138	153	13.6
19	CBD ca	62	M	3.8	4.2	55	105	253
20	Hilar CC	66	M	4.4	3.5	61	89	497
21	CBD ca	66	F	3.2	3.9	24	33	70.0
22	CBD ca	77	M	3.8	1.9	62	183	41.0
23	Hilar CC	90	F	3.7	4.3	84	54	341

CC, Cholangiocarcinoma; ca, cancer; CBD, common bile duct; AST, aspartate aminotransferase; ALT, alanine aminotransferase; CA 19-9, carbohydrate antigen 19-9.

**Table 2:** Top 14 Proteins identified as more abundant in cholangiocarcinoma than control

Protein	Gene	MW (kDa)	Average Spectral Counts		Log (Fold)	P value
			control	cancer		
Immunoglobulin kappa light chain		23	0.00	2.27	27.50	0.03
Apolipoprotein B	APOB	516	0.00	14.56	25.25	0.08
Inter-alpha-trypsin inhibitor heavy chain H4	ITIH4	101	0.00	5.31	24.98	0.06
Apolipoprotein E	APOE	36	0.00	3.47	24.89	0.03
Mucin-5B	MUC5B	596	0.00	5.67	24.10	0.08
Inter-alpha-trypsin inhibitor heavy chain H1	ITIH1	101	0.00	3.54	23.84	0.06
Apolipoprotein A-IV	APOA4	45	0.00	2.30	23.05	0.18
Intercellular adhesion molecule 1	ICAM1	58	0.00	2.36	22.78	0.06
Complement C7	C7	94	0.00	1.78	21.56	0.29
Complement C5	C5	188	0.00	1.50	20.61	0.44
Apolipoprotein C-III	APOC3	13	0.00	1.00	20.61	0.19
Albumin	ALB	69	45.00	422.61	5.55	0.045
Antithrombin-III	SERPINC1	53	1.00	6.25	4.25	0.03
Apolipoprotein A-II	APOA2	11	1.00	2.45	2.20	0.26

**Table 3:** Proteins identified as significantly increased in cholangiocarcinoma than control

Protein	Gene	MW (kDa)	Average Spectral Counts		Log (Fold)	P value
			control	cancer		
Immunoglobulin kappa light chain		23	0.00	2.27	27.50	0.033
Apolipoprotein E	APOE	36	0.00	3.47	24.89	0.026
Albumin	ALB	69	45.00	422.61	5.55	0.045
Apolipoprotein A-I	APOA1	31	2.50	17.28	4.70	0.02
Antithrombin-III	SERPINC1	53	1.00	6.25	4.25	0.03
Α1-antitrypsin	SERPINA1	47	17.75	61.33	3.70	0.037
Serotransferrin	TF	77	7.33	45.78	3.70	0.002
Immunoglobulin heavy constant mu	IGHM	49	3.00	6.78	3.25	0.045
Immunoglobulin J chain	JCHAIN	18	2.00	2.82	3.22	0.022
Complement C4-A	C4A	193	3.00	22.06	2.72	0.021
Complement C3	C3	187	11.67	39.61	2.32	0.017
